# *In vitro* models of the gut-liver axis: what we’ve learned and what remains to be built

**DOI:** 10.3389/fimmu.2026.1858289

**Published:** 2026-06-03

**Authors:** Jong Hwan Sung, Raehyun Kim

**Affiliations:** 1 Department of Chemical Engineering, Hongik University, Seoul, Republic of Korea; 2 Department of Biological and Chemical Engineering, Hongik University, Sejong, Republic of Korea

**Keywords:** gut-liver axis, *in vitro* model systems, inter-organ crosstalk, microphysiological system, multi-organ model systems

## Abstract

The gut-liver axis maintains metabolic homeostasis and immune regulation through continuous bidirectional communication, and its dysregulation contributes to a range of metabolic, inflammatory, and immune-mediated diseases. Integrated gut-liver axis model systems offer unique tools for dissecting these complex interactions by isolating individual variables that are difficult to disentangle *in vivo*, while allowing flexible experimental controls over them. Here, we review advances in gut-liver axis models from static co-cultures to microfluidic systems and their applications in pharmacokinetic and mechanistic studies. We identify underexplored areas, including metabolite-mediated gut-liver crosstalk, immune-mediated interorgan communication, and disease-specific modeling, and outline technical challenges to achieving physiologically faithful and reliable integrated platforms. By addressing these challenges, gut-liver axis models will contribute to a mechanistic understanding of gut-liver pathobiology that is difficult to achieve through clinical studies, animal models, or individual organ systems alone.

## 1 Introduction

The gut and liver are anatomically and functionally linked through continuous bidirectional communication essential for metabolic homeostasis and immune regulation. Nutrients, microbial metabolites, bacterial products (e.g., LPS), and gut-primed immune cells reach the liver via the portal vein, where they are metabolized, detoxified, or cleared. In turn, liver-derived mediators, including bile acids, cytokines, and acute-phase proteins, regulate gut barrier function and immune responses. This bidirectional communication, termed the gut-liver axis, plays a critical role in systemic physiology and contributes to the pathogenesis of metabolic, inflammatory, and neoplastic diseases (1).

Integrated gut-liver axis models that functionally couple both organs have emerged in recent years, initially driven by pharmacokinetic applications. These platforms, ranging from static co-cultures to microfluidic organ-on-chip systems, enable the investigation of inter-organ communication that cannot be captured in isolated systems. This review evaluates the contributions of integrated gut-liver models to mechanistic research, identifies underexplored areas including metabolite-mediated crosstalk, immune-mediated communication, and disease-specific modeling, and outlines key challenges and future directions.

## 2 Technological evolution of *in vitro* gut-liver axis models

The key to creating an *in vitro* model of the gut-liver axis is in recapitulating the dynamic bidirectional interactions between the gastrointestinal tract and the liver. Over the years, a range of diverse *in vitro* model systems have been developed to realize the gut-liver axis. Here, we categorize these models based on their underlying technology and describe their pros and cons.

### 2.1 Static co-culture model or conditioned media

In the simplest form, a directly mixed co-culture of gut-derived cells and liver-derived cells in a single cell culture well is possible (2), although only rarely employed because it does not provide compartmentalized, directional communication between the two entities, and neglects key physiological aspects such as the gut epithelium and vasculature. A more conventional approach uses a Transwell format, with gut cells typically cultured in the upper compartment on a semipermeable membrane and liver cells in the lower compartment (3), reflecting drug absorption across the gut epithelium and delivery to the liver via the portal vein. The opposite arrangement is also possible, depending on the direction of the interaction being pursued, for example, to examine the effect of the hepatocytes on the enterocytes (4). Another simple approach uses ‘conditioned media’ (4), in which culture media is transferred between cell types to deliver signaling molecules. This method can be expanded to other cell types, such as adipocytes, to study interactions among the gut, liver, and adipose tissue (5).

### 2.2 Microfluidic-based models

Beyond compartmentalized co-culture in static conditions, recapitulating the gut-liver axis requires capturing the dynamics of molecular transport between the two organs. The gastrointestinal tract and the liver are connected via the portal vein and biliary tract, and the mode of material exchange between the two is mainly achieved by convection through the vessels and diffusion in the tissue area. In this regard, microfluidic systems offer distinct advantages, since microfluidic channels can mimic the transport of molecules via vascular convection. Utilizing the principles of fluid dynamics, the size of microfluidic channels can be designed so that the convection velocity and residence time closely match those of *in vivo* counterparts (6, 7). Incorporating 3D hydrogel-based cell culture can further enhance physiological fidelity by adding a diffusion barrier that mimics the transport within *in vivo* tissue (8, 9).

**Figure 1:**
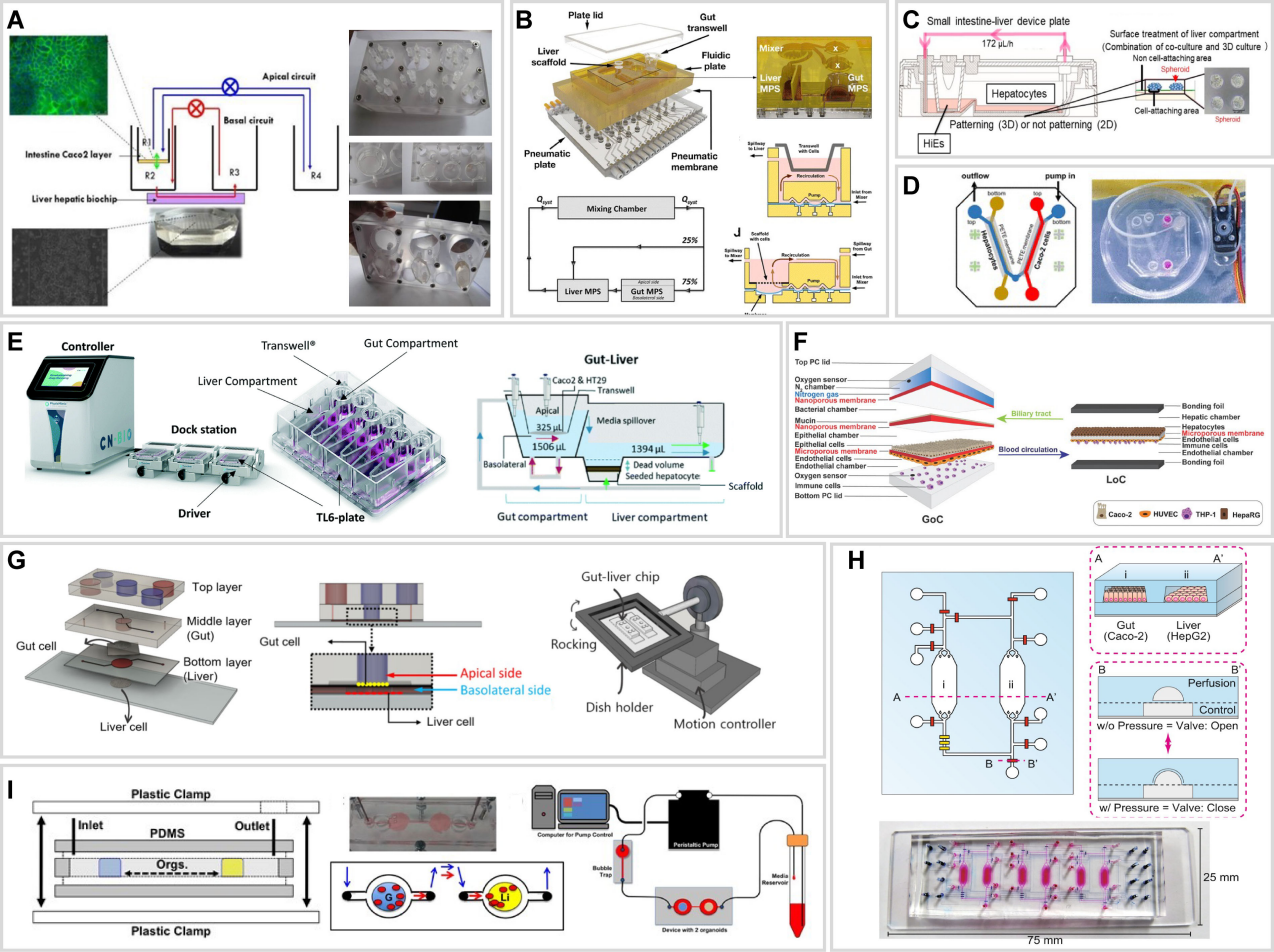
Representative gut-liver-on-a-chip platforms developed for studying first-pass metabolism, drug pharmacokinetics, and gut-liver axis interactions. **(A)** Microfluidic platform coupling intestinal and liver compartments for evaluating ADME processes, used to study paracetamol metabolism; adapted with permission from (12), Copyright 2014, Wiley Periodicals, Inc. **(B)** Integrated gut and liver microphysiological system designed for quantitative *in vitro* pharmacokinetic studies; adapted from (14) under the terms of the Creative Commons Attribution 4.0 International License (CC BY 4.0), Copyright 2017, The Authors. **(C)** Perfusing small intestine–liver microphysiological system device; adapted from (21) under the terms of the Creative Commons Attribution 4.0 International License (CC BY 4.0), Copyright 2023, The Authors. **(D)** Perfluoropolyether-based gut-liver-on-a-chip for evaluating first-pass metabolism and oral bioavailability of drugs; adapted with permission from (22), Copyright 2024 American Chemical Society. **(E)** Gut-liver-on-a-chip device combined with mechanistic modeling for pharmacokinetic study of mycophenolate mofetil; adapted from (23) under the terms of the Creative Commons Attribution 3.0 Unported License (CC BY 3.0), Copyright 2022, The Authors, published by the Royal Society of Chemistry. **(F)** Organ-on-chip platform simulating drug metabolism along the gut-liver axis; adapted from (24) under the terms of the Creative Commons Attribution (CC BY) License, Copyright 2024, The Authors. **(G)** *In vitro* hepatic steatosis model based on a gut-liver-on-a-chip; adapted with permission from (18), Copyright 2021, American Institute of Chemical Engineers. **(H)** Integrated gut-liver-on-a-chip platform as an *in vitro* human model of non-alcoholic fatty liver disease; adapted from (17) under the terms of the Creative Commons Attribution 4.0 International License (CC BY 4.0), Copyright 2023, The Authors. **(I)** Reductionist metastasis-on-a-chip platform for *in vitro* tumor progression modeling and drug screening; adapted with permission from Ref. (33), Copyright 2016, Wiley Periodicals, Inc.

Early examples of microfluidic gut-liver systems, such as those by Choe et al., and Lee et al., co-cultured gut and liver cells in vertically arranged chambers separated by semipermeable membranes and connected by perfusion channels (10, 11). This setting builds on the Transwell format by adding perfusion, enabling convective molecular transport between the gut and liver compartments while maintaining experimental simplicity. Leclerc et al., developed fluidic circuits to achieve convective transport between the gut and liver chambers (**Figure 1A**) (12, 13). Tsamandouras et al. developed an integrated gut and liver fluidic system for quantitative *in vitro* pharmacokinetic studies using a circulating common medium (**Figure 1B**) (14). These systems were primarily developed to observe the first-pass metabolism and predict pharmacokinetics, but some studies focused more on the effect of fluidic shear stress and molecular signaling on the inter-tissue crosstalk (15, 16). Recent examples have focused more on specific disease models, such as fatty liver disease and hepatic damage by ethanol (17–19), which we discuss in more detail in Section 3.

### 2.3 Organoid or primary cell culture-based gut-liver platforms

Incorporating organoid or primary cell cultures into microfluidic systems can offer a physiological model that combines improved biological functionality with a fluidic environment enabling inter-organ crosstalk. Skardal et al. reported an integrated multi-organoid system incorporating tissue organoids of liver, heart, lung, vasculature, testis, colon, and brain to test the toxicities of a panel of drugs that were recalled by the FDA (20). More recently, Sakai et al. reported using human iPS cell-derived small intestinal epithelial cells and cryopreserved human primary hepatocytes in 3D culture within a microfluidic platform (**Figure 1C**) (21). Gene expression levels of major hepatic metabolizing enzymes, such as CYP1A2 and CYP3A4, and transepithelial electrical resistance (TEER) of the small intestinal epithelial cultures were increased, implying improved physiological relevance while allowing gut-liver crosstalk. These attempts hold great promise for achieving physiologically realistic gut-liver interactions, but adapting organoid and primary cell culture models to microfluidic platforms will require further optimization and validation.

## 3 Demonstrated applications of gut-liver axis models

### 3.1 First-pass metabolism and toxicity models

Early *in vitro* models of the gut-liver axis primarily focused on reproducing first-pass metabolism, that is, the absorption through the gut epithelium and subsequent hepatic metabolic processes. Despite varying in structure, these systems share a fundamental concept: gut and liver cell cultures, whether in 2D or 3D, are fluidically connected within a microfluidic system to enable molecular transport and reactions. Proof-of-concept systems demonstrated that drugs can be absorbed through the gut barrier, and subsequently metabolized by hepatic cells (10–12).

More recent examples include the work by Wang et al., where a gut-liver chip was developed to evaluate the first-pass metabolism and oral bioavailability of midazolam (MDZ) (22). Notable features include the incorporation of perfluoropolyether (PFPE) to minimize the unwanted sorption of drugs, and an enterohepatic single-passage system to simplify the analysis, as opposed to a recirculation system (**Figure 1D**). Milani et al. studied the absorption and conversion of mycophenolate mofetil, a prodrug, to its active form, mycophenolic acid, and further metabolism to glucuronide metabolite (**Figure 1E**) (23). Combining experimental measurements with mechanistic modeling allowed estimation of pharmacokinetic parameters, including clearance (CL) and permeability (P). Lucchetti et al, described a multi-organ-on-a-chip (MOoC) for simulating the metabolism of irinotecan, a colon cancer drug, along the gut-liver axis (**Figure 1F**) (24). A notable feature of their work was the incorporation of the gut microbiome into their system, which revealed the role of *Escherichia coli* in producing toxic metabolites.

### 3.2 Disease models

Dysregulation of the gut-liver axis is associated with various diseases, including non-alcoholic fatty liver disease (NAFLD) and inflammatory bowel disease (IBD), often associated with metabolic dysfunction. The progression of such diseases involves impairment of gut epithelium and liver function, elevated immune responses, alterations in gut microbiota, and the release of toxic metabolites (25, 26). While early *in vitro* models of the gut-liver axis primarily targeted reproducing the first-pass metabolism, more recent efforts have shifted toward disease modeling that capture complex interactions between multiple components (gut, liver, immune system, microbiota, vasculature, etc.). Lee et al. reported one of the first gut-liver chip models of fatty liver disease, adapting a previously developed plaform to reproduce fatty acid absorption and its accumulation in liver cells (**Figure 1G**) (27). This system was further developed to incorporate immune cells and adipocytes (18, 28–30).

Yang et al. reported an integrated gut-liver-on-a-chip platform as a model of NAFLD, where gut and liver cell lines, Caco-2 and HepG2, were cultured in a microfluidic device with an interconnected, closed-circulation loop (**Figure 1H**) (17). Following the treatment with free fatty acids (FFA), phenotypic and genotypic changes were evaluated. The first-pass metabolism of ethanol and consequent tissue damage, such as hyperpermeability and stromal injury, were observed using an intestine-liver axis-on-chip (19). A simple Transwell format was used to co-culture gut and liver cells in a study aimed to mimic the crosstalk between the intestine and liver during NAFLD progression (31). Exposing Caco-2 cells in the apical chamber of the Transwell insert to LPS and/or FFA increased the permeability of the gut barrier, ApoB expression, and triglyceride secretion in the basolateral media, resulting in hepatic damage from oxidative stress and aldehyde derivative production.

Another example of a disease model where the gut-liver axis plays a central role is cancer metastasis, as colorectal cancer has a known tendency to metastasize preferentially to the liver, primarily due to the vascular connection between the two organs (32). A ‘metastasis-on-a-chip’ was developed to track colon cancer cells migrating from a 3D gut construct to a downstream liver construct (**Figure 1I**) (33). In this chip, key migratory events in the metastatic cascade, including the growth of metastatic tumor foci, dissemination from the gut construct, entry into circulation, and subsequent colonization of the liver construct, were observed. In a subsequent study by the same researcher, a multi-site metastasis-on-a-chip was developed to assess the metastatic preference of cancer cells by connecting the gut construct with the liver, lung, and endothelial constructs (34). Representative microfluidic gut-liver axis models discussed above are summarized in **Table 1**.

**Table 1:** Examples of microfluidic gut-liver axis models. .

No.	Gut source	Liver source	Drugs tested or disease modeled	Application	Notable chip features	References
1	Caco-2 cell line	Cell line (ex: HepG2)	Paracetamol, apigenin	First-pass metabolism	Gravity-driven flow	(10, 11)
2	Caco-2 cell line	Cell line (ex: HepG2)	Phenacetin, paracetamol	First-pass metabolism	Pump-operated system	(12, 13)
3	Genome-edited CYP/UGT expressing Caco-2	Primary hepatocytes	Midazolam (MDZ)	First-pass metabolism	perfluoropolyether (PFPE)	(22)
4	Caco-2/HT29 co-culture	Primary hepatocytes	Mycophenolate mofetil	First-pass metabolism	PhysioMimix (from CN Bio)	(23)
5	Caco-2 cell line	HepRG cell line	Irinotecan	First-pass metabolism	Gut bacterium, immune and vascular endothelial cells included	(24)
6	Caco-2 cell line	HepG2 cell line	NAFLD	Disease model	Immune cells, adipocytes	(18, 27–30)
7	Caco-2 cell line	HepG2 cell line	NAFLD	Disease model	Single-cell profiling	(17)
8	Human intestinal myofibroblasts, Caco-2	HepG2	Ethanol-induced hepatic damage	First-pass metabolism/disease model	3D cell culture models	(19)
9	Caco-2 cell line	HepG2 cell line	NAFLD	Disease model	Transwell system	(31)
10	HCT-116, SW480	HepG2	Cancer metastasis	Disease model	3D gut and liver lung, endothelial constructs	(33, 34)

The significance of the gut-liver axis in the progression of various diseases is now well established, including non-alcoholic fatty liver disease, alcoholic liver disease, primary sclerosing cholangitis (PSC) (1), IBD (35), and cancer metastasis (32). With the continuous advancement of multi-organ-on-a-chip systems that enable interactions among various organs (36–39), further development of such systems is likely to offer valuable insights into the mechanisms underlying these diseases.

## 4 Underexplored aspects of gut-liver communication

While the gut-liver axis models have demonstrated value in specific applications (section 3), major mechanistic aspects of inter-organ communication remain underexplored. Here, we identify areas where integrated models could provide unique insights but are underdeveloped.

### 4.1 Metabolite-mediated gut-liver crosstalk

Metabolite-mediated communication through the portal circulation is a central feature of gut-liver axis physiology, but remains insufficiently represented in integrated *in vitro* systems. Short-chain fatty acids (SCFAs) are among the most extensively studied gut-derived metabolites with anti-inflammatory and metabolic effects demonstrated in intestinal epithelial and immune cells, and therapeutic potential for hepatic steatosis (40–42). *In vivo*, SCFAs activate intestinal G protein-coupled receptors (GPR41, GPR43, and GPR109A), stimulating gut hormone release that regulates hepatic lipogenesis and gluconeogenesis (43–45). However, integrated gut-liver models have not yet captured organ-specific SCFA production, transport, and metabolic effects, in part due to challenges in co-culturing obligate anaerobic microbes with aerobic host tissues, although exogenous SCFA supplementation in integrated systems could enable investigation of organ-specific metabolic responses without requiring live microbial communities.

Bile acids activate intestinal FXR, inducing FGF19 secretion, which suppresses hepatic CYP7A1 through FGFR4 signaling, completing a feedback loop that regulates bile acid synthesis and broader metabolic processes (46, 47). While individual components of this pathway have been modeled in isolation, integrated systems have not recapitulated the full regulatory loop, primarily due to technical barriers such as engineering functional biliary secretion. Partial reconstruction, for example, through exogenous bile acid administration, could enable investigation of FXR activation and downstream hepatic metabolic regulation without requiring complete enterohepatic circulation.

### 4.2 Immune-mediated gut-liver communication

Immune regulation is fundamental to gut-liver homeostasis, yet remains only partially reconstructed in current models. Intestinal immune cells continuously sample microbial and dietary antigens, which are transported to the liver via portal circulation along with gut-primed immune cells (48, 49). Hepatic antigen-presenting cells, including Kupffer cells, dendritic cells, sinusoidal endothelial cells, and hepatocytes, maintain immune tolerance through low-costimulatory antigen presentation, while innate and innate-like lymphocytes (NK, NKT, and MAIT cells) contribute to pathogen surveillance (50, 51). Disruption of this tolerogenic environment contributes to immune-mediated liver disease (52, 53).

Although recent platforms have begun incorporating immune cells, physiologically relevant immune integration remains limited. Most current implementations rely on exogenous cytokine stimulation or the addition of circulating immune cells, which capture generalized inflammatory responses but do not reproduce tissue-specific immune programming or tolerogenic regulation. For example, a gut-liver-brain microphysiological system incorporating CD4+ T and Th17 cells demonstrated the feasibility of introducing circulating immune components into multi-organ platforms but did not reconstruct tissue-resident immune populations or organ-specific immune functions (54, 55). Critical immune features that remain to be recapitulated include active sentinel sensing at the gut barrier, trafficking of immune cells and inflammatory signals between organs, and tolerogenic filtering within the liver.

### 4.3 Gut-liver pathobiology and disease-specific modeling

Despite growing recognition that gut-liver crosstalk drives disease initiation and progression, the mechanistic understanding remains limited. Clinical and animal studies face inherent constraints, including patient heterogeneity, environmental variability, and species differences in immune regulation, metabolism, and microbiome composition, which complicate causal inference (56–59). Integrated gut-liver *in vitro* systems offer a complementary approach by enabling controlled manipulation of individual factors in human cells, yet disease-specific integrated models remain largely unexplored (60, 61).

Cirrhosis exemplifies this bidirectional interaction. Compromised intestinal barrier function permits the translocation of microbial products, contributing to hepatic inflammation and dysfunction, including bile acid dysregulation, portal hypertension, and immune impairment (62). This further compromises intestinal function, establishing a self-reinforcing pathological cycle (62). Strong clinical associations between intestinal dysfunctions and hepatic pathology have also been observed in PSC and autoimmune hepatitis (AIH). IBD occurs in 60-80% of PSC patients (63, 64), and a 20-year follow-up study reported increased IBD incidence among AIH patients and greater cirrhosis prevalence in AIH-IBD patients compared to AIH alone (65). A Mendelian randomization study further suggested a causal contribution of IBD to AIH risk (66). Integrated gut-liver systems could enable controlled investigation of these mechanisms by isolating individual factors and defining their contributions to disease initiation and progression.

## 5 Technical challenges and paths forward

The gaps identified above highlight biological frontiers that remain to be explored. Pursuing these opportunities, however, introduces distinct technical and engineering challenges that must be addressed to improve mechanistic insight, translational relevance, and experimental utility.

### 5.1 The gut microbiome integration

As noted in Section 4.1, most models have relied on exogenous bacterial products rather than live bacteria or live microbial communities. However, specific microbial species, not their metabolites, can causally contribute to liver disease, as demonstrated by endotoxin-producing *Enterobacter cloacae* or high-alcohol-producing *Klebsiella pneumoniae* (67, 68). Moreover, microbial abundance and metabolic activity are regulated by inter-microbial interactions, including nutrient competition and crossfeeding, which cannot be captured by exogenous products alone (69, 70).

Recent advances in gut model systems featuring a steep oxygen gradient across the epithelium have enabled co-culture of obligate anaerobes with intestinal epithelium (71–76). However, maintaining stable microbial communities over extended periods remains challenging, particularly when integrated with liver compartments. Despite these obstacles, microbiome integration represents a major opportunity given its central role in metabolic, inflammatory, and immune-mediated liver disease.

### 5.2 Immune dynamics

Bridging the gaps in immune components identified in Section 4.2 requires direct incorporation of immune cells, yet practical barriers remain. Most existing platforms rely on exogenous cytokine supplementation, which does not fully recapitulate immune surveillance, direct cell-cell interactions, or coordinated immune responses. Restoring these functions requires tissue-resident immune cells, which enable microbial sensing, cytokine relay, and maintenance of tissue homeostasis, and circulating immune cells that mediate inter-organ crosstalk through selective recruitment and migration.

A major barrier is sourcing immune cells with appropriate tissue specificity and phenotypic stability. IPSC-derived immune cells offer the most physiologically faithful solution, but remain technically demanding and resource-intensive. Primary immune cells offer greater accessibility but are limited by lifespan and donor variability. Surrogate cell lines, such as THP-1-derived macrophages or dendritic-like cells, provide reproducible immune sensing and cytokine signaling, enabling mechanistic investigation despite limited tissue-specific programming (77). Similarly, endothelial cell lines with sinusoidal features (such as SK-HEP-1) can partially model hepatic immune interfaces (78). These complementary approaches define a spectrum of immune integration strategies balancing fidelity and feasibility.

### 5.3 Enhancing the physiological relevance through design optimization

Complete replication of *in vivo* gut-liver axis complexity is neither feasible nor necessary. Rather, the goal is to incorporate key design features that preserve inter-organ communication and tissue-specific functions.

Physiological relevance within each compartment can be enhanced through several approaches. A staged cell sourcing strategy - initial optimization using robust cell lines followed by primary cells or iPSCs - can balance fidelity with reproducibility. Extracellular matrix selection should reflect tissue-specific requirements, with basement membrane components to support epithelial polarity and hepatocyte differentiation, and interstitial matrices to facilitate interactions among parenchymal, endothelial, and immune cells (79). Oxygen gradients deserve particular attention, as they are essential for supporting anaerobic gut microbiota while preserving oxidative metabolism in host cells. Physiologically relevant flow and shear stress should also be considered for their effects on cellular phenotypes and metabolic functions (80).

Inter-organ communication depends on a set of design parameters. Compartment geometry, volume ratios, and fluidic connections determine the kinetics of molecular transport between gut and liver modules, and should be designed to approximate physiologically relevant transit times and dilution factors (61, 81). Physiologically relevant scaling of different organs — matching relative organ sizes, residence times, and metabolic capacities rather than absolute dimensions — requires careful consideration of various, inter-connected sets of parameters, and often it is extremely difficult to achieve the optimal set of parameters due to physical and experimental restrictions (11, 81). Still, these engineering considerations define the biochemical and physical context in which gut-liver crosstalk occurs, and careful optimization of these parameters will be critical for advancing integrated platforms toward mechanistic utility. Physiologically-based pharmacokinetic (PBPK) modeling and *in vitro*-to-*in vivo* extrapolation (IVIVE) provide a framework for interpreting the data obtained from novel *in vitro* models and predicting human response (82).

### 5.4 Validation, translation, and in silico integration

A regulatory shift toward reducing animal use is now underway globally, reflected in the US Food and Drug Administration (FDA)’s roadmap to reduce animal testing in preclinical safety studies (83), parallel European Medicines Agency (EMA) activity to define regulatory acceptance criteria for microphysiological systems under its 3Rs framework (84), and Japan’s AMED-MPS program advancing microphysiological systems toward regulatory application (85). These developments facilitate the adoption of new approach methodologies (NAMs), including organ chips, organoids, computer modeling, and artificial intelligence (AI). For NAMs, qualification is granted for a context of use rather than for the model itself, and each context of use requires a separate qualification process, making fit-for-purpose validation the operative standard (86). Aligned with this, validation should reflect intended biological function rather than direct equivalence to *in vivo* systems. Establishing a stable, physiologically relevant baseline state that reflects homeostasis, together with coherent and directional responses to defined perturbations such as microbial products or inflammatory stimuli, provides the evidence supporting context-specific qualification. Standardizing protocols and readouts will improve reproducibility and facilitate cross-platform comparisons that regulatory acceptance ultimately requires.

Incorporating in silico models (digital twins) will provide an analytical and quantitative framework for interpreting the experimental results, designing the experiments, and extrapolating the obtained data to predict *in vivo* responses in humans (i.e., IVIVE). Recent rapid advances in AI and machine learning (ML) technologies will also facilitate the integration of *in vitro* and in silico models. Along with *in vitro* models including organoids and microphysiological systems (organ-on-a-chip), in silico models such as AI/ML predictive models and quantitative systems pharmacology (QSP) models are explicitly identified as NAMs in the FDA Roadmap (83). Ultimately, coupling fit-for-purpose validation with in silico integration will determine whether gut-liver axis models progress from descriptive research tools to qualified, decision-ready platforms within this evolving regulatory landscape.

## 6 Conclusions

Gut-liver axis models have demonstrated mechanistic utility in specific applications while revealing substantial gaps in metabolite-mediated interorgan communication, immune dynamics, and chronic disease modeling. Advancing these frontiers requires addressing challenges in integrating the microbiome and the immune system, the two essential components for most pathobiological questions. Pragmatic approaches, including staged cell sourcing and fit-for-purpose validation, can balance physiological relevance with experimental feasibility. As technologies mature, gut-liver axis models will increasingly bridge reductionist cell culture and whole-organism studies, enabling mechanistic insights inaccessible through other experimental approaches.
